# Degradation Paradigm of the Gut Hormone, Pancreatic Polypeptide, by Hepatic and Renal Peptidases

**DOI:** 10.1210/en.2016-1827

**Published:** 2017-03-09

**Authors:** Joyceline Cuenco, James Minnion, Tricia Tan, Rebecca Scott, Natacha Germain, Yiin Ling, Rong Chen, Mohammad Ghatei, Stephen Bloom

**Affiliations:** 1 Section of Investigative Medicine, Division of Diabetes, Endocrinology and Metabolism, Imperial College London, London SW7 2AZ, United Kingdom; 2 Department of Endocrinology, Centre Hospitalier Universitaire de Saint-Etienne, Saint-Etienne 42100, France

## Abstract

Pancreatic polypeptide (PP) is a gut hormone that acts on Y4 receptors to reduce appetite. Obese humans display a reduced postprandial increase in PP and remain fully sensitive to the anorectic effects of exogenous PP. The utility of PP as an anti-obesity treatment is limited by its short circulating half-life. Insight into the mechanisms by which PP is degraded could aid in the design of long-acting PP analogs. We investigated the role of peptidases in PP degradation to determine whether inhibition of these enzymes enhanced PP plasma levels and bioactivity *in vivo*. Dipeptidyl peptidase IV (DPPIV) and neprilysin (NEP) were two peptidase found to cleave PP. Limiting the effect of both peptidases improved the *in vivo* anorectic effect of PP and PP-based analogs. These findings suggest that inhibiting the degradation of PP using specific inhibitors and/or the design of analogs resistant to cleavage by DPPIV and NEP might be useful in the development of PP as an anti-obesity pharmacotherapy.

## Teaser

The degradation sites of pancreatic polypeptide (PP) by kidney and liver tissue preparations were investigated and used to create longer lasting anorectic analogs of PP.

Obesity is a global health crisis. The only effective long-term treatment is bariatric surgery. However, the limited financial and specialist medical resources mean that bariatric surgery is not a viable therapy for the entire obese population (1, 2). Additionally, gastrointestinal, metabolic, and nutritional complications of surgery have been frequently described in the published data (3). Therefore, a need exists to develop novel, nonsurgical therapies. It is well established that the secretion of gut hormones in response to food intake can regulate appetite by acting directly on the brain or indirectly through vagal afferents (4), or by a combination of both. An analog of the gut hormone glucagon-like peptide 1 (GLP-1) has recently been approved for use in weight management, demonstrating the value of appropriating endogenous systems for the treatment of obesity (5). Altering gut hormone levels could, therefore, be useful in the treatment or prevention of obesity and in the improvement of the associated consequences such as insulin resistance.

Pancreatic polypeptide (PP) is an amidated 36-amino-acid peptide from the PP-fold family. It is released from F cells in the islets of Langerhans cells of the pancreas in response to meal ingestion (6, 7). The magnitude of PP secretion is proportional to caloric intake (8, 9), and its release can also be stimulated by other hormones such as cholecystokinin and ghrelin and in response to adrenergic activation during hypoglycemia or exercise (10). PP has a high affinity for the Y4 receptor, and its effects on food intake are mediated via this receptor (11).

Intravenous and peripheral administration of PP reduces food intake in mice and humans (12–14). Overexpression of PP in mice produces a hypophagic and thin phenotype, suggesting that chronic exposure to PP does not lead to attenuation of the anorectic effect. The administration of anti-PP antiserum reverses this phenotype (15).

Obese subjects demonstrate a blunted postprandial PP response (16, 17), and subjects with anorexia nervosa have higher circulating levels of PP (18) and an exaggerated postprandial PP response (19). Paradoxically, Prader-Willi patients who are obese and experience uncontrollable hyperphagia have high basal levels of PP. However, they still exhibit the blunted PP response to feeding (17), and infusion of PP significantly reduces acute food intake in patients with Prader-Willi syndrome (9). This robust anorectic effect suggests that PP has potential as an anti-obesity treatment.

Administration of PP to humans is not associated with adverse side effects such as nausea, unlike GLP-1 analogs and, as such, is potentially a useful agent for the regulation of food intake and the treatment of obesity. PP is a relatively small peptide (4.2 kDa) and is therefore susceptible to a number of degradative enzymes and other eliminating mechanisms. PP consequently has a short circulating half-life of approximately 7 minutes in humans (6). This short half-life limits the use of native PP as a practical obesity treatment. To the best of our knowledge, the mechanisms for the degradation of PP that result in its short half-life have not been previously reported.

The major sites of proteolytic degradation of peptides are the kidney and liver, where proteolytic enzymes are found in high concentrations (20, 21). The present study evaluated the hepatic and renal degradation of PP and its modification to produce an analog with extended bioefficacy. We investigated whether a couple of proteases known to cleave other gut hormones (22, 23), dipeptidyl peptidase IV (DPPIV) and neprilysin (NEP), are involved in the degradation of PP. NEP is abundantly present on renal membranes, and PP levels are known to be elevated in patients with renal failure (24). Circulating DPPIV is increased in obese patients and correlates negatively with circulating levels of PP (18). To investigate the significance of these proteases, we modified the sequence of PP to remove known sites of enzymatic degradation and used the specific protease inhibitors sitagliptin (a DPPIV inhibitor) and phosphoramidon (a NEP inhibitor). Finally, we used our knowledge of the sites of peptide degradation to produce an analog of PP that would be a more suitable anti-obesity treatment.

## Materials and Methods

### Peptides and enzymes

Human PP and PP analogs were purchased from Bachem Ltd. (St. Helens, UK). Human recombinant NEP (EC3.4.24.11) was purchased from R&D Systems (Abingdon, UK). Human DPPIV, the DPPIV inhibitor sitagliptin, and the metallopeptidase inhibitor phosphoramidon, were purchased from Sigma-Aldrich (Dorset, UK).

### Degradation assays using rat liver microsomes and renal brush border membranes

Rat renal brush border (RBB) membranes were prepared using a method of homogenization and centrifugation, as described previously (25). RBB (1 mg/mL) was incubated with PP (2 nmol) for 10 or 60 minutes in digest buffer (300 mM mannitol, 12 mM HEPES; pH 7.4) at 37°C. Rat liver microsomes (RLMs) were also prepared as described previously (26). Peptides were incubated with RLM in digest buffer (0.10 M Tris-HCl buffer; pH 7.5) with a total volume of 110 μL containing 2 nmol of peptide and 1 mg/mL (10 µg/reaction) RLM at 37°C. Digest reactions were terminated by the addition of 5 μL of 10% volume-to-volume ratio of trifluoroacetic acid, and the membranes were removed by centrifugation at 21,000*g* (Sigma 3K18, rotor catalog no. 12348; Sigma-Aldrich) for 5 minutes at room temperature. The supernatant was analyzed using high-performance liquid chromatography (HPLC) and matrix-assisted laser desorption/ionization-time of flight (MALDI-ToF) mass spectrometry, as previously described (27). The incubation time and RLM concentration were selected based on studies that had demonstrated differences in susceptibility to degradation between peptides.

### Degradation assays using purified DPPIV and NEP enzymes

PP (2 nmol) was incubated with or without 10 mU DPPIV (digest buffer, 100 mM Tris-HCl; pH 8) or 200 ng of recombinant human NEP (digest buffer, 25 mM Tris-HCl, 0.1 M NaCl; pH 8) in a volume of 120 µL of buffer, for a concentration of l6.7 µM PP, at 37°C (28). The reactions were terminated at the stated time point by the addition of 5 µL of 10% volume-to-volume ratio trifluoroacetic acid. Samples were then centrifuged at 21,000*g* (Sigma 3K18; rotor catalog no. 12348; Sigma-Aldrich) for 5 minutes at room temperature, and 100 µL of the supernatant was immediately analyzed using HPLC and mass spectrometry.

### Y4 receptor binding assay

Transfected HEK293 cells overexpressing the mouse Y4 receptor were prepared and binding assays performed as previously described (29). Isolated cell membranes (protein concentration, 1 to 2 µg/mL) were incubated for 90 minutes in siliconized polypropylene tubes together with ^125^I-PP [1 kBq (100 pM)] and a dose response of unlabeled competing peptides at 4°C in binding buffer [20 mM HEPES (pH 7.4), 1 mM MgCl_2_, 5 mM CaCl_2_, 0.1% bovine serum albumin, and protease inhibitors (0.2 mM phenylmethylsulfonyl fluoride, 0.1 mM Diprotin A, and 10 µM phosphoramidon ( all from Sigma-Aldrich)] in a final assay volume of 0.5 mL. PP was iodinated as previously described (8) using ^125^I from GE Healthcare Life Sciences (Amersham, UK). Pelleted membranes were washed with 5 mL ice-cold assay buffer, and the membranes were centrifuged at 15,874*g* for 3 minutes at 4**°**C, as described, to separate bound and free label. Bound radioactivity was measured using a *γ*-counter (NE 1600; NE Technology Ltd, Reading, UK). Specific binding was calculated as the difference between the amount of ^125^I-PP bound in the absence (total binding) and presence of 5 μM unlabeled competing peptide (nonsaturable binding). All curves were performed with points in triplicate. Half maximal inhibitory concentration values were calculated using Prism, version 5.01 (GraphPad Software Inc., San Diego, CA) using a three-parameter logistic equation for the nonlinear regression fit: Y = bottom + (top − bottom)/1 + 10^(LogEC_50_ − X), where EC_50_ is the half maximal effective concentration.

### MALDI-ToF mass spectrometry

PP digests were analyzed using MALDI-ToF mass spectrometry. In brief, freeze-dried samples were reconstituted using 100 μL solution A (60% AcN, 40% water, and 0.1% trifluoroacetic acid). The matrix (10 mg/mL α-cyano-4-hydrocinnanic acid in solution A) was applied to the sample plate (0.5 μL), followed immediately by the sample (0.5 μL), and the plate was air-dried. The mass spectrometer (Shimadzu Axima-CFR; Shimadzu, Milton Keynes, UK) was set to a positive linear mode. Before data acquisition from the samples, the instrument was calibrated using a mixture of five peptides across the mass range 1000 to 6000. Peptide fragments resulting from the cleavage of PP were identified (accounting for monoisotopic mass allowance) using the program FindPept (available at: http://www.expasy.ch/tools/findpept.html).

### *In vivo* studies

#### Animals

All animal procedures were approved by the British Home Office, under the United Kingdom Animal (Scientific Procedures) Act 1986 (project license, 70/6402). Adult male C57/BL6 mice (Harlan, Wyton, UK) weighing 20 to 25 g were maintained in individual cages under controlled temperature (21°C to 23°C) and light (12-hour light/dark cycle, with lights on at 7:00 am). The mice had *ad libitum* access to water and normal chow RM1 (Special Diet Services, Devon, UK), unless stated otherwise. For acclimatization, the mice were regularly handled and given 2 sham subcutaneous (SC) and/or intraperitoneal (IP) injections of saline. Before each feeding study, the mice were fasted overnight, and the injections were performed during the early light phase (8:00 to 10:00 am). The mice were stratified by body weight.

### Anorectic effect of enzyme-resistant analogs of PP

To investigate the anorectic effects of DPPIV-resistant analogs, the mice were stratified into treatment groups and administered a SC injection (maximum volume, 100 µL) of vehicle, PP, PP3-36, PP2-36, or PP-Ala0 (n = 7 of 9). Food intake was measured at 1, 2, 4, and 8 hours after injection to compare the peptides with short half-lives (PP and its endogenous degradation product, PP3-36) and, in a separate experiment, at 24 hours after injection for peptides with modifications (PP-Ala0 and PP2-36, using PP as the comparator). A PP analog with global modifications for enzyme resistance (PP-x) was also investigated and administered as a SC injection (maximum volume, 100 µL) at a high and low dose (n = 9). Food intake was measured at 1, 2, 4, and 8 hours after injection.

### Effect of DPPIV and NEP inhibition on anorectic effect of PP

To investigate the effect of inhibition of NEP by phosphoramidon, the mice were stratified into four treatment groups and administered an IP injection followed by a SC injection 15 minutes later (maximum volume, 50 µL, SC) of (1) vehicle (water for injections) IP, followed by saline SC; (2) phosphoramidon (5 mg/kg) IP, followed by saline SC; (3) vehicle IP, followed by PP SC (150 nmol/kg); or (4) phosphoramidon (5 mg/kg) IP, followed by PP SC (150 nmol/kg; n = 10). This dose of phosphoramidon was sufficient to inhibit NEP activity (30). Food intake was measured at 1, 2, 4, 8, 24, and 48 hours after injection. The same study design was then repeated using 6 mg/kg of the DPPIV inhibitor, sitagliptin, which has previously been shown to work through IP administration (31).

### Effect of phosphoramidon on plasma levels of PP and PP analogs in mice

The mice were divided into four treatment groups according to body weight. A protocol similar to that used for the phosphoramidon feeding study was used, with an IP injection, followed by a SC injection 15 minutes later (maximum volume, 50 µL for each injection) of phosphoramidon (0, 7, 20, and 60 mg/kg IP), followed by PP (150 nmol/kg SC in saline; n = 4 to 6; all groups at 45 and 90 minutes). The mice were killed by increasing carbon dioxide asphyxiation at 45 and 90 minutes after the SC injection. Additional mice (n = 2) underwent overnight fasting and were culled at 0 minutes after injection of the control substances (saline SC and vehicle IP) for measurement of the baseline levels of circulating PP. The blood was immediately removed via cardiac puncture and centrifuged at 6000 rpm (Sigma 3K18; 12348 rotor; Sigma-Aldrich) for 10 minutes. The plasma was stored at −20°C until analysis. The plasma samples were assayed using an established in-house PP RIA (8). The anti-PP antibody was elevated in rabbit against human PP and recognized PP (and other truncated forms) but not in other PP-fold peptides or gastrointestinal peptides. ^125^I-PP was prepared using the iodogen method and purified using HPLC. The assay was performed in duplicate in a total volume of 700 µL of 0.06 M phosphate buffer (pH 7.35) containing 0.3% bovine serum albumin. The samples were incubated for 3 days at 4°C before separation of free and antibody-bound ^125^I-PP using dextran-coated charcoal. The sensitivity was ≤5 fmol/tube. The intra-assay coefficient of variation was 6%.

### Statistical analysis

All data are presented as the mean ± standard error of the mean. *In vitro* data were analyzed using the unpaired *t* test, with the assumption that the data were normally distributed. Interval food intake data and plasma-level data were analyzed using one- or two-way analysis of variance with Dunnett’s or Bonferroni’s *post hoc* test (GraphPad Prism, version 5.03; GraphPad Software). In all cases, *P* ≤ 0.05 was considered to indicate statistical significance.

## Results

### Characterization of RBB and RLM-induced peptide fragments

**Figure 1.**
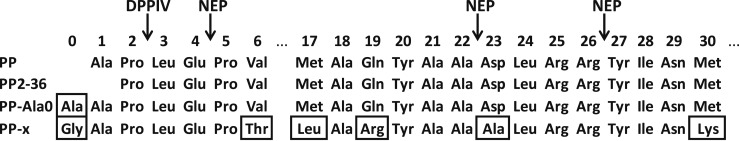
Sequence of PP and primary sites of hydrolysis. The cleavage sites for each enzyme are denoted by arrows. The sequence of PP analogs is included for comparison.

MALDI-ToF mass spectrometry was used to determine the mass of peptide fragments derived after incubation of PP with RBB or RLMs. A number of PP fragments were detected after incubation of PP with RBB and appeared in a time-dependent manner. PP incubated with RBB for 10 minutes generated molecules with a molecular weight corresponding to PP3-36 and PP6-33. In contrast, 60-minute incubations resulted in molecules with a molecular weight corresponding to PP3-36, PP3-35, PP3-29, PP5-31, and PP23-36, consistent with cleavage sites at Pro2-Leu3, Glu4-Pro5, and Ala22-Asp23, respectively (Table 1). A 60-minute incubation of PP with RLM resulted in molecules with a molecular weight corresponding to PP1-35, PP3-36, and PP8-26. These results were consistent with the cleavage sites Pro2-Leu3 and Arg26-Tyr27. Cleavage at Pro2-Leu3 was observed after DPPIV incubation, and other putative cleavage sites at Glu4-Pro5, Ala22-Asp23, and Arg26-Tyr27 were observed after incubation with NEP (Table 1). A summary of putative enzymatic cleavage sites is shown in Fig. 1.

**Table 1.** **Breakdown Products of PP by Tissue Preparations or Recombinant Enzymes, as Characterized by MALDI-ToF**

Enzyme/Membrane	Incubation Time (min)	MALDI-TOF (MW)	Putative Sequence of Peptide Products		
			Sequence	MW	Cleavage Sites
RBB	10	4183	PP_1–36_	4183	—
		4015	PP_3–36_	4015	Pro_2_-Leu_3_
		3259	PP_6–33_	3259	Pro_5_-Val_6_
					Arg_33_-Pro_34_
			PP_10–36_	3259	Gly_9_-Asp_10_
	60	4184	PP_1–36_	4183	—
		4015	PP_3–36_	4015	Pro_2_-Leu_3_
		3853	PP_3–35_	3851	Pro_2_-Leu_3_
					Arg_35_-Tyr_36_
		3259	PP_6–33_	3259	Pro_5_-Val_6_
					Arg_33_-Pro_34_
			PP_10–36_	3259	Gly_9_-Asp_10_
		3097	PP_3–29_	3096	Pro_2_-Leu_3_
					Asn_29_-Met_30_
			PP_5–31_	3098	Glu_4_-Pro_5_
					Leu_31_-Thr_32_
		1867	PP_23–36_	1867	Ala_22_-Asp_23_
RLM	60	4185	PP_1–36_	4183	—
		4055	PP_1–35_(2Met-O)	4052	Arg_35_-Tyr_36_
		4016	PP_3–36_	4015	Pro_2_-Leu_3_
		2106	PP_8–26_	2104	Tyr_7_-Pro_8_
					Arg_26_-Tyr_27_
		2068	PP_14–30_	2070	Thr_13_-Pro_14_
					Met_30_-Leu_31_
		2030	PP_1–19_	2028	Gln_19_-Tyr_20_
DPPIV	120	4014	PP_3–36_	4015	Pro_2_-Leu_3_
		4112	PP_2–36_	4112	—
		4255	PP-Ala0	4255	—
NEP	120	1724	PP_12–26_	1721	Asn_11_-Ala_12_
					Arg_26_-Tyr_27_
			PP_7–22_	1727	Val_6_-Tyr_7_
					Ala_22_-Asp_23_
		1242	PP_1–12_	1242	Ala_12_-Thr_13_
		647	PP_5–10_	647	Glu_4_-Pro_5_
					Asp_10_-Asn_11_
Trypsin	15	2875	PP_1–26_	2874	Arg_26_-Tyr_27_
		1481	PP_26–36_	1483	Arg_25_-Arg_26_
		1165	PP_27–35_	1163	Arg_26_-Tyr_27_
					Arg_35_-Tyr_36_
			PP_26–34_		Arg_25_-Arg_26_
					Pro_34_-Arg_35_
			PP_28–36_		Tyr_27_-Ile_28_
			PP_16–25_	1166	Glu_15_-Gln_16_
					Arg_25_-Arg_26_

Abbreviation: MW, molecular weight.

### Effect of *N*-terminal modifications on DPPIV-mediated degradation *in vitro*

DPPIV-resistant analogs of PP were designed through *N*-terminal extension (PP-Ala0) and *N*-terminal truncation (PP2-36) to eliminate the DPPIV target sequence of proline at position 2 of the molecule. Incubation of each analog with DPPIV resulted in single peaks as determined by MALDI-ToF mass spectrometry, which were consistent with the theoretical masses of PP-Ala0 and PP2-36 and suggested no degradation by DPPIV (Table 1).

### Effect of *N*-terminal modifications on anorectic effect of PP analogs

**Figure 2.**
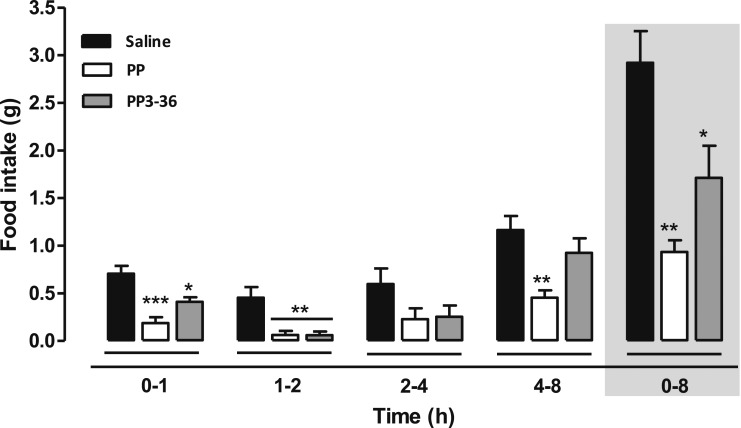
Effect of SC administration of PP and PP3-36 (150 nmol/kg) on food intake in C57/BL6 mice fasted overnight at 0- to 1-, 1- to 2-, 2- to 4-, and 4- to 8-hour intervals after injection and cumulatively at 0 to 8 hours (shaded region). PP and PP3-36 were compared with saline using one-way analysis of variance with Bonferroni’s *post hoc* test: *P ≤ 0.05, **P ≤ 0.01, ***P ≤ 0.001 (n = 7).

PP and the DPPIV degradation product, PP3-36, significantly reduced food intake at 0- to 1- and 1- to 2-hour intervals after injection (0- to 1-hour food intake, *P* < 0.05 for PP3-36 and *P* < 0.001 for PP vs saline; and 1- to 2-hour food intake, *P* < 0.001 vs saline; Fig. 2). Only PP significantly reduced food intake in the 4- to 8-hour interval (*P* < 0.01 vs saline). Both PP and PP3-36 significantly reduced food intake compared with vehicle control compared with during the 0- to 8-hour period (saline, 2.92 ± 0.29 g; vs PP, 0.93 ± 0.10 g, *P* < 0.01; vs PP3-36, 1.71 ± 0.29 g, *P* < 0.05). Overall, no statistically significant difference was found in the anorectic effect between PP and PP3-36. Nevertheless, PP3-36 did appear to have an attenuated anorectic effect compared with PP (Fig. 2).

**Figure 3.**
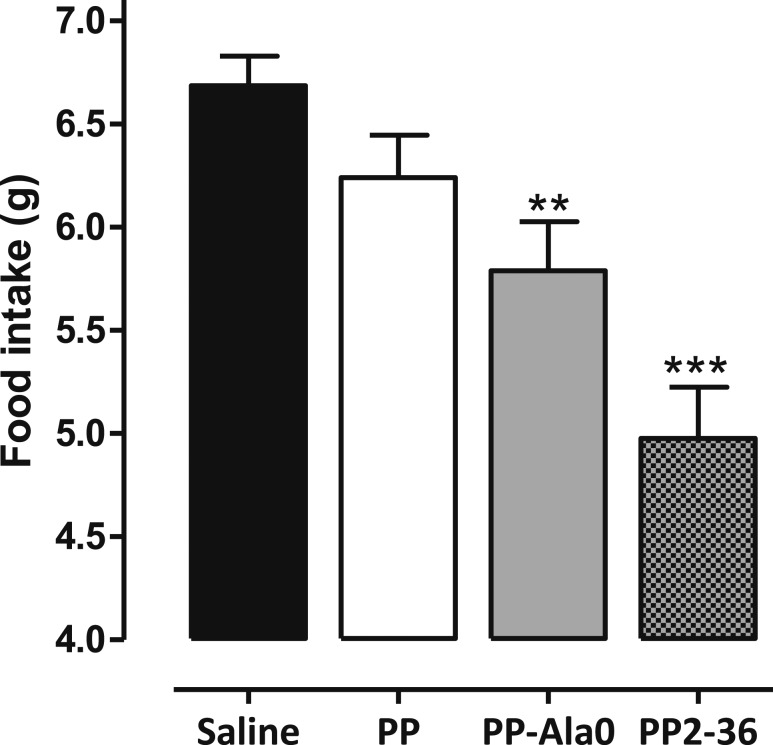
Effect of SC administration of PP, PP-Ala0, and PP2-36 (150 nmol/kg) on food intake in C57/BL6 mice fasted overnight at 0 to 24 hours after injection. PP, PP-Ala0, and PP2-36 were compared with saline using one-way analysis of variance with Dunnett’s *post hoc* test: **P ≤ 0.01, *** *P* ≤ 0.001 (n = 7).

The receptor affinity of DPPIV-resistant analogs PP2-36 and PP-Ala0 was similar to that of PP (half maximal inhibitory concentration, 0.31, 0.35, and 0.26 nM, respectively; Table 2). PP2-36 and PP-Ala0 produced a statistically significant inhibition of food intake over 24 hours (PP2-36, *P* ≤ 0.05; and PP-Ala0, *P* ≤ 0.01 vs saline), but PP did not (Fig. 3).

**Table 2.** **Receptor Binding Affinity for PP and Analogs for Y4 Receptor**

Peptide	IC_50_ (nM)
PP	0.26 ± 0.04
PP2-36	0.31 ± 0.06
PP-Ala0	0.35 ± 0.05
PP-x	0.064 ± 0.005

Data presented as mean ± standard error of the mean of three to five separate experiments.

Abbreviations: IC_50_, half maximal inhibitory concentration.

### Effect of sitagliptin and phosphoramidon on the anorectic effect of PP

**Figure 4.**
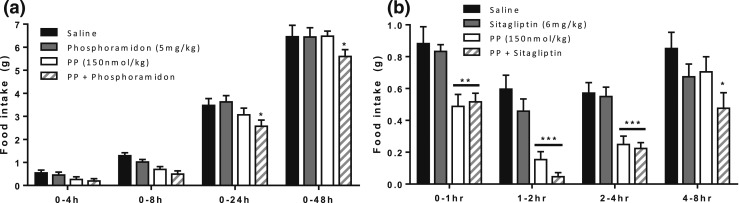
Effect of SC administration of (a) PP (150 nmol/kg), phosphoramidon (5 mg/kg), and PP plus phosphoramidon; and (b) sitagliptin (6 mg/kg) and PP plus sitagliptin on food intake in C57BL/6 mice fasted overnight. Cumulative food intake shown for (a) ≤48 hours after injection and (b) intervals ≤8 hours after injection. Significance analyzed using two-way analysis of variance with Bonferroni’s *post hoc* test: *P ≤ 0.05, **P ≤ 0.01, ***P ≤ 0.001 (n = 9).

Treatment alone with 5 mg/kg phosphoramidon did not cause a substantial reduction of food intake at any of the intervals measured [Fig. 4(a)]. PP combined with phosphoramidon significantly reduced food intake in the 8- to 48-hour period compared with saline (saline at 24 hours, 3.47 ± 0.22 g; vs PP plus phosphoramidon at 24 hours, 2.60 ± 0.18 g; *P* < 0.01). PP plus phosphoramidon significantly decreased food intake compared with PP alone at 48 hours after injection [PP at 48 hours, 6.52 ± 0.14 g vs PP plus phosphoramidon at 48 hours, 5.63 ± 0.21 g; *P* < 0.01; Fig. 4(a)]. In the study of sitagliptin, treatment with 6 mg/kg sitagliptin alone did not cause a substantial reduction of food intake at any of the intervals measured [Fig. 4(b)]. At a dose of 150 nmol/kg, PP significantly reduced food intake for ≤4 hours after injection (saline at 4 hours, 2.07 ± 0.11 g vs PP at 4 hours, 0.91 ± 0.04 g; *P* < 0.001). PP combined with sitagliptin significantly reduced food intake for ≤8 hours after injection compared with saline [saline at 8 hours, 2.92 ± 0.14 g; vs PP plus sitagliptin at 8 hours, 1.28 ± 0.12 g; *P* < 0.05; Fig. 4(b)].

### Effect of phosphoramidon on circulating levels of PP and PP-Ala0

**Figure 5.**
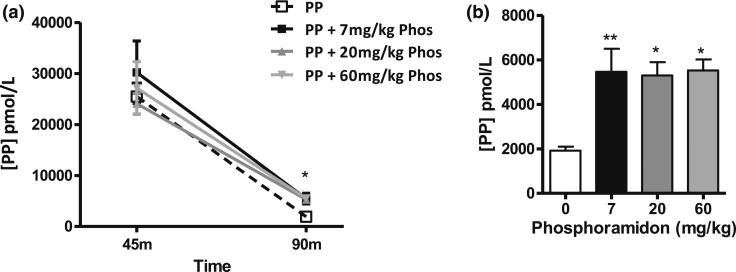
Plasma levels of PP after a single SC injection of PP at 150 nmol/kg to male C57BL/6 mice combined with a single IP injection of phosphoramidon (Phos) at 7 mg/kg, 20 mg/kg, and 60 mg/kg. Blood samples were collected at (a) 45 and (b) 90 minutes after injection (n = 2 to 4). Data presented as the mean plasma levels of PP ± standard error of the mean as determined by radioimmunoassay (lower limit of detection, 70 pmol/L). Significance analyzed using (a) two-way analysis of variance or (b) one-way analysis of variance, both with Bonferroni’s *post hoc* test: *P ≤ 0.05, **P ≤ 0.01.

Phosphoramidon was administered at 7, 20, or 60 mg/kg via an IP injection, 15 minutes before SC administration of PP (150 nmol/kg). The plasma levels of PP were measured at 45 and 90 minutes after the SC peptide injection. At 45 minutes after injection, the plasma levels of PP did not differ significantly between PP administered alone or combined with any dose of phosphoramidon [Fig. 5(a)]. At 90 minutes after injection [Fig. 5(b)], the plasma levels of PP were significantly greater when combined with any dose of phosphoramidon compared with PP given alone (PP alone, 1920 ± 134 pmol/L vs PP plus phosphoramidon [at 7, 20, and 60 mg/kg], 5460 ± 807 pmol/L, 5297 ± 461 pmol/L, and 5525 ± 348 pmol/L, respectively).

**Figure 6.**
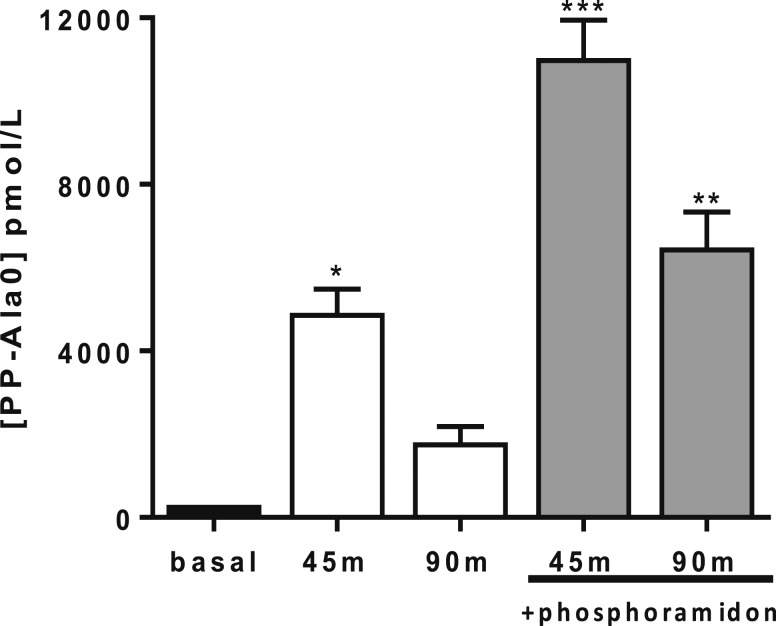
Plasma levels of PP-Ala0 at 45 and 90 minutes (m) after a single SC injection (150 nmol/kg) to male C57BL/6 mice (n = 6), with and without a prepeptide IP injection of phosphoramidon (20 mg/kg). Basal plasma samples were collected from naïve mice (n = 2). Data presented as mean plasma levels of peptide ± standard error of the mean as determined by radioimmunoassay (lower limit of detection, 40 pmol/L). Significance analyzed using two-way analysis of variance with Bonferroni’s *post hoc* test: *P ≤ 0.05, **P ≤ 0.01, or ***P ≤ 0.001.

The DPPIV-resistant PP analog, PP-Ala0, was also coadministered with phosphoramidon. The mice had significantly greater plasma levels of PP-Ala0 at both 45 and 90 minutes after injection when administered with phosphoramidon compared with PP-Ala0 given alone (45 minutes, PP-Ala0, 4916 ± 480 pmol/L vs PP-Ala0 plus phosphoramidon, 11,034 ± 703 pmol/L; 90 minutes, PP-Ala0, 1809 ± 319 pmol/L vs PP-Ala0 plus phosphoramidon, 6484 ± 722 pmol/L; Fig. 6) and compared with the basal levels of PP-Ala0 immunoreactivity.

### Effect of global modifications on anorectic effect of PP

**Figure 7.**
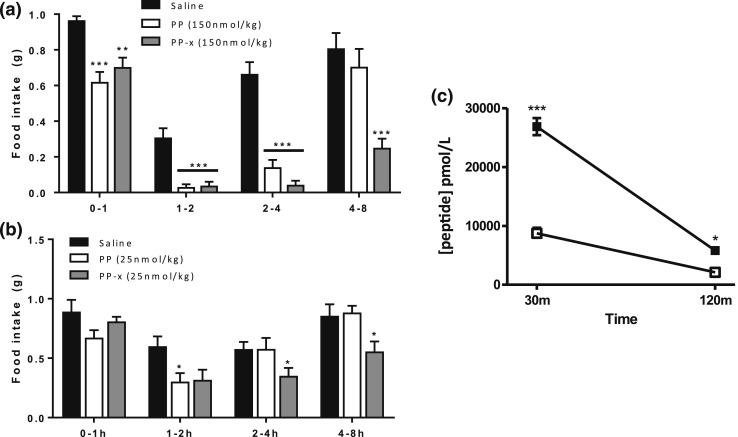
Effect of SC administration of PP and PP-x at (a) 150 nmol/kg and (b) 25 nmol/kg on food intake in C57BL/6 mice fasted overnight (n = 9). PP and PP-x were compared with saline using one-way analysis of variance with Bonferroni’s *post hoc* test. (c) Plasma peptide levels at 30 minutes and 2 hours after a SC injection of PP (open squares) and PP-x (closed squares) administered at 1000 nmol/kg (n = 4). Peptide concentrations were measured using radioimmunoassay. Significance was measured using two-way analysis of variance with Bonferroni’s *post hoc* test: *P ≤ 0.05, **P ≤ 0.01, ***P ≤ 0.001.

A PP analog was designed that accounted for certain modifications to protect against DPPIV and NEP (PP-x; Fig. 1). PP and PP-x were administered at a high dose (150 nmol/kg) and low dose (25 nmol/kg). At the high dose [Fig. 7(a)], PP and PP-x significantly reduced food intake compared with the saline control group for ≤4 hours after injection (*P* ≤ 0.001). However, only PP-x significantly reduced food intake for ≤8 hours after injection (saline at 8 hours, 2.75 ± 0.06 g; vs P-x at 8 hours, 1.04 ± 0.09 g; *P* < 0.001). At the low dose [Fig. 7(b)], PP significantly reduced food intake only at the 1-to 2-hour interval (*P* ≤ 0.05). However, again only PP-x significantly reduced food intake for ≤8 hours after injection (saline at 8 hours, 2.93 ± 0.13 g; vs P-x at 8 hours, 1.98 ± 0.14 g; *P* < 0.01). Cumulative food intake at the 0- to 8-hour interval showed a difference between the anorectic effect of PP and PP-x, but the difference did not reach statistical significance (PP at 8 hours, 2.36 ± 0.18 g; vs P-x at 8 hours, 1.98 ± 0.14 g; *P* = NS).

### Effect of global modifications on circulating plasma levels of PP-x

The mice were given a SC injection of either PP or PP-x (100 nmol/kg). The mice were killed at 30 or 120 minutes after injection. The peptide levels of PP at 30 minutes after injection reached 8737 ± 744 pmol/L and had significantly decreased by 120 minutes to 2112 ± 224 pmol/L [Fig. 7(c)]. The peptide levels for PP-x at 30 minutes after injection reached 26,862 ± 1026 pmol/L and had significantly decreased at 2 hours to 5810 ± 215 pmol/L. All peptide levels had decreased to 22% to 24% at 2 hours after injection compared with the peptide levels at 30 minutes; however, the plasma levels of PP-x were significantly greater than the PP levels at both 30 and 120 minutes after injection (*P* ≤ 0.001).

## Discussion

We investigated the effects of tissue membrane preparations on the degradation of PP and found a putative role for DPPIV and NEP in the degradation of PP. These findings could aid in the development of long-acting protease-resistant PP analogs.

PP is susceptible to a number of degradative enzymes and other eliminating mechanisms, resulting in a short circulating half-life (6). Thus, the clearance rate is a particularly limiting factor for using PP as an anti-obesity therapy. The major sites of proteolytic degradation of peptides in general are the kidney and liver, where proteolytic enzymes are found at high concentrations. *In vitro* degradation studies using cell membrane extracts from the kidney (RBB) and liver (RLMs) identified some residues in the breakdown of PP. These enzymatic cleavage patterns were confirmed using purified enzymes *in vitro*, and *in vivo* studies were used to confirm a possible mechanism. We also investigated whether substitutions of residues identified as prone to proteolytic cleavage would result in improved circulating plasma levels and, therefore, pharmacological action.

Both RBB and RLMs had degradative effects on PP. RBB cleaved PP at Pro2-Leu3, Glu4-Pro5, and Ala22-Asp23, and RLMs cleaved PP at Pro2-Leu3 and Arg26-Tyr27.

Fragment PP3-36 is a product of the enzymatic action of DPPIV and was observed in digests with both RLMs and RBB, in agreement with known sites of DPPIV expression (32, 33). DPPIV cleaves the first two amino acids of the *N*-terminal of peptides up to approximately 80 amino acids long. The preferred substrate of DPPIV is X-Pro-X (where X is any of the naturally occurring mammalian amino acid, except for Pro). Therefore, the *N*-terminal sequence of PP (Ala1-Pro2-Leu3) would be expected to be degraded by DPPIV. MALDI-TOF mass spectrometry confirmed PP was degraded by DPPIV to PP3-36. As expected, PP5-36 was not observed, because Pro5 at the P3′ cleavage subsite should not be degraded further by DPPIV.

DPPIV alters the Y receptor affinity of PYY, another member of the PP-fold family, when it is cleaved from PYY1-36 to PYY3-36; thus, it becomes a Y2r-preferring agonist from a Y1r agonist. In contrast, degradation of PP to PP3-36 by DPPIV had no substantial reduction in affinity to its endogenous Y4 receptor. The bioefficacy of the PP3-36 was tested by comparing its ability to inhibit food intake in mice compared with the parent molecule. Surprisingly, PP3-36 was able to reduce food intake, although its anorectic effects were attenuated. Although no statistically significant difference was found between the groups treated with PP and PP3-36, PP3-36 appeared less potent than PP compared with the control group, particularly at later time points.

Two strategies to achieve resistance to DPPIV were to extend the *N*-terminal to create PP-Ala0 or to truncate the *N*-terminus to produce PP2-36. Both of these analogs obviated degradation by DPPIV, as confirmed by MALDI-ToF. The biological activity of both DPPIV-resistant PP analogs were tested in acute feeding studies in mice. PP-Ala0 and PP2-36 produced significantly greater inhibition of food intake than PP over 24 hours, confirming the beneficial effect of DPPIV resistance.

Many of the cleavage sites identified by the RLM and RBB digests were potential sites for cleavage by NEP, a cell-surface metallopeptidase, that typically targets hydrophobic residues and is present in RBB (34, 35) and liver (36, 37) at high concentrations. To assess the role of NEP in the ability of PP to reduce food intake (bioefficacy), PP was administered with and without the potent NEP inhibitor, phosphoramidon (22, 38). The DPPIV inhibitor, sitagliptin, was also coadministered with PP to confirm the role of DPPIV. Neither sitagliptin nor phosphoramidon alone had a substantial effect on food intake, in agreement with previous studies (27, 30). In fasted animals, the intrinsic levels of PP are very low. Limiting the degradation of the low levels of endogenous hormone (*e.g.,* using enzyme inhibitors alone) is therefore unlikely to have a substantial effect on food intake, when a potent physiological stimulus to eat is present because of high levels of ghrelin. The physiological relevance of these degradation pathways is likely to be more relevant postprandially. In this situation, the degradation of satiety hormones such as PP is important such that appetite is switched back on relatively soon after eating, because, from an evolutionary context, it is important to continue to eat when food is abundant to prevent starvation in times when food is no longer available. When exogenous sources of PP were administered at pharmacological doses, together with a DPPIV or an NEP inhibitor, food intake was significantly reduced compared with the control group and compared with administration of PP alone. A limitation of these inhibitor studies was that enzyme inhibitors could also influence the degradation of endogenous hormones, which might additionally influence appetite, although no evidence of this nonspecific effect was demonstrated in the group treated with inhibitor alone compared with saline. An additional approach to assess the physiological importance of NEP was to measure the plasma levels of PP in mice when PP was administered with and without phosphoramidon. The plasma levels of PP when administered with phosphoramidon remained elevated for longer than when PP was administered alone. Together, these data suggest that NEP is partly responsible for the clearance of PP from the circulation.

A DPPIV-resistant analog of PP was also administered with phosphoramidon to test whether NEP contributed to the prolonged anorectic action of the DPPIV-resistant PP analog, PP-Ala0. Although the peptide plasma levels of native PP at 90 minutes after injection were almost 80% less than that observed at 45 minutes after injection, the PP-Ala0 levels remained elevated for longer, with only 60% of the peptide cleared between 45 and 90 minutes after injection. Thus, providing DPPIV resistance to the PP molecule aids in reducing the degradative effects of NEP. A possible explanation is that Pro2, found in native PP, forms part of the hydrophobic “zipper” in the core of the PP-fold structure and is the main interaction partner for Tyr27 (39). The loss of Pro2 after DPPIV degradation causes destabilization of the PP-fold structure and could generally make the molecule more unstable. This might cause it to be more susceptible to proteolytic degradation by other peptidases such as NEP. Thus, designing analogs of PP with resistance to both DPPIV and NEP degradation could be important in producing longer lasting analogs of PP.

PP-x was the final analog of PP designed and was designed with global modifications against multiple enzymatic cleavage sites. PP-x was able to reduce food intake for a longer period compared with native PP, suggesting it is more resistant to degradation in circulation. This idea was supported by the plasma levels measured for both peptides. Although both peptides demonstrated substantial reductions in plasma levels at 120 minutes compared with their respective peptide levels at 30 minutes, the absolute value of PP-x at 120 minutes was still significantly greater than that of PP (almost threefold) and might be sufficient to maintain the anorectic effects of Y4 receptor agonist (40).

In conclusion, pharmacotherapy with gut hormones might be a more cost-effective treatment strategy for obesity than bariatric surgery. Subjects with constitutional thinness, characterized by a resistance to body weight gain, are often viewed as an anti-obesity model. This population displays high levels of the gut hormone PYY (41) and an exaggerated postprandial PYY response after overconsumption of calories from fat (42). Thus, gut hormones (*e.g.,* GLP-1 and PYY) can contribute to weight management that might eventually halt the development of obesity. Increasing endogenous levels of PP by decreasing degradation, increasing the half-life, or increasing bioavailability might provide a plan for treating obesity, potentially as a part of a combination drug therapy approach. The data we have presented support a role for DPPIV and NEP in the degradation of PP. The design methods we adopted led to the development of a DPPIV- and NEP-resistant analog of PP (43) with improved pharmacokinetics. Our results support the strategy of understanding the degradative patterns of native peptides and rationally targeting areas susceptible to breakdown to produce a more efficacious treatment. This strategy, in general, might prove useful in the quest for effective anti-obesity therapy.
